# Reduced microbial diversity of the nasopharyngeal microbiome in household contacts with latent tuberculosis infection

**DOI:** 10.1038/s41598-023-34052-8

**Published:** 2023-05-05

**Authors:** Cinthya Ruiz-Tagle, Juan A. Ugalde, Rodrigo Naves, Rafael Araos, Patricia García, María Elvira Balcells

**Affiliations:** 1 Departamento de Enfermedades Infecciosas del Adulto, Escuela de Medicina, Pontificia Universidad Católica de Chile, Santiago, Chile; 2 Center for Bioinformatics and Integrative Biology, Facultad de Ciencias de La Vida, Universidad Andrés Bello, Republica 330, Santiago, Chile; 3 Instituto de Ciencias Biomédicas, Facultad de Medicina, Universidad de Chile, Santiago, Chile; 4 Instituto de Ciencias E Innovación en Medicina, Facultad de Medicina Clínica Alemana, Universidad del Desarrollo, Santiago, Chile; 5 Advanced Center for Chronic Diseases (ACCDiS), Santiago, Chile; 6 Laboratorio de Microbiología, Departamento de Laboratorios Clínicos, Escuela de Medicina, Pontificia Universidad Católica de Chile, Santiago, Chile

**Keywords:** Microbiome, Tuberculosis

## Abstract

The upper respiratory tract is an obliged pathway for respiratory pathogens and a healthy microbiota may support the host's mucosal immunity preventing infection. We analyzed the nasopharyngeal microbiome in tuberculosis household contacts (HHCs) and its association with latent tuberculosis infection (TBI). A prospective cohort of HHCs was established and latent TBI status was assessed by serial interferon-γ release assay (IGRA). Nasopharyngeal swabs collected at baseline were processed for 16S rRNA gene sequencing. The 82 participants included in the analysis were classified as: (a) non-TBI [IGRA negative at baseline and follow-up, no active TB (n = 31)], (b) pre-TBI [IGRA negative at baseline but converted to IGRA positive or developed active TB at follow-up (n = 16)], and (c) TBI [IGRA positive at enrollment (n = 35)]. Predominant phyla were *Actinobacteriota*, *Proteobacteria*, *Firmicutes* and *Bacteroidota*. TBI group had a lower alpha diversity compared to non-TBI (p_adj_ = 0.04) and pre-TBI (p_adj_ = 0.04). Only TBI and non-TBI had beta diversity differences (p_adj_ = 0.035). Core microbiomes’ had unique genera, and genus showed differential abundance among groups. HHCs with established latent TBI showed reduced nasopharyngeal microbial diversity with distinctive taxonomical composition. Whether a pre-existing microbiome feature favors, are a consequence, or protects against *Mycobacterium tuberculosis* needs further investigation.

## Introduction

Tuberculosis (TB) is an airborne infectious disease caused by the pathogenic bacteria *Mycobacterium tuberculosis* (Mtb). About a quarter of the global population is infected with Mtb^1^. The susceptibility to acquiring this infection depends on intensity^2^ and duration of exposure^3^. Host characteristics such as sex, age, genetics, immune status, nutrition, and environmental and social factors (crowding, poor ventilation, alcohol, smoking, and occupational risk) increase the risk of TB^4,5^; still, individuals with no known risk factors account for a large number of new cases.

The microbiota inhabiting our body provides a milieu that benefits the entire host-microbe system, influencing host homeostasis and immune response^6,7^, with each person having distinct microbe communities. A healthy microbiota supports the host’s immunity against infections by promoting mucosal barrier function, competing with pathogens, and priming immune responses. At the same time, its disruption can increase the risk of disease^8,9^. The upper respiratory tract is colonized by specialized resident bacterial assemblages, presumably preventing potential pathogens from overgrowing and disseminating toward the lungs, thereby functioning as gatekeepers to respiratory health^10^. The characteristics of the nasal and nasopharyngeal microbiome from infected and uninfected individuals have been shown to differ in upper respiratory tract infections such as sinusitis, otitis media, influenza, RSV, and SARS-CoV-2^11,12^.

There is less evidence about the nasopharyngeal microbiota composition in lower respiratory tract bacterial infections. Two studies reported comparatively lower alpha diversity indices of microbial diversity in children with pneumonia^13,14^, while another study in adults did not. Nonetheless, the three studies reported that a more diverse microbiota is found in the nasopharynx of healthy individuals^13–15^. In addition, the experimental nasal inoculation of *Streptococcus pneumoniae* was shown to induce a shift in the nasopharyngeal microbiota of healthy adults correlating with the establishment of the bacterial carriage^16^.

In TB pathogenesis, the role of the respiratory tract microbiome remains largely unexplored. Thus, we assessed the nasopharyngeal microbiome in a prospective cohort of individuals exposed to Mtb to ascertain if there are differences in microbiome composition or abundance between individuals that acquired or not a latent TB infection, aiming to gain further insight into TB pathogenesis and susceptibility.

## Results

### Clinical and demographic data

From the enrolled cohort of 231 HHCs, 155 participants (67%) had complete follow-up and fulfilled inclusion/exclusion criteria for microbiome analysis (Supplementary Fig. S1). As per our classification, 44% were classified as TBI (n = 69), 41% as non-TBI (n = 63), and 15% as pre-TBI (n = 23).

From the 155 samples selected for 16S sequencing, 82 (53%) remained after the ASVs filtering process, with a considerable loss due to the initial low microbial biomass of the nasopharyngeal swabs collected samples. For analysis, 31 (38%) samples pertained to the Mtb uninfected (non-TBI), 16 (19%) from those acquiring a new Mtb infection on follow-up (pre-TBI), and 35 (43%) from HHCs displaying Mtb infection since the baseline evaluation (TBI). The demographic and clinical characteristics of HHCs included in the analysis are summarized in Table 1. Overall, 58.5% were female, the mean age was 34.5 (± 13.7) years, and all were residents in Chile of Hispanic/Latino descent, primarily migrants from Peru (58.5%). Individuals with TBI were slightly older than non-TBI and pre-TBI (p = 0.013). The comparison of the demographic and clinical characteristics of the entire cohort (composed of individuals whose samples were excluded, unsuccessfully sequenced, and successfully sequenced/analyzed) did not reveal substantial differences between groups and is summarized in Supplementary Table S1.

**Table 1:** Clinical and epidemiological characterization of TB contacts included in the analysis (n = 82).

	Non-TBI (n = 31)	Pre-TBI (n = 16*)	TBI (n = 35†)	p-value
Female sex (n, %)	16 (51.6%)	10 (62.5%)	22 (62.9%)	0.611
Mean age, years (SD)	32.5 (11.8)	27.9 (12.0)	39.2 (14.7)	0.013
Countries, total (n, %)				0.222
Peru	17 (54.8%)	9 (56.3%)	22 (62.9%)	
Chile	9 (29.0%)	1 (6.3%)	8 (22.9%)	
Other Latin American countries	5 (16.1%)	6 (37.5%)	5 (14.3%)	
Tobacco smoker (n, %)	8 (25.8%)	3 (18.8%)	10 (28.6%)	0.683
Self-reported viral respiratory infections in last month (n, %)	10 (32.3%)	5 (31.3%)	13 (37.1%)	0.883
Any antibiotic use—Last 6 months (n, %)	7 (22.6%)	1 (6.3%)	5 (14.3%)	0.386
Probiotic or vitamin use—Last 3 months (n, %)	7 (22.6%)	3 (18.8%)	2 (5.7%)	0.125
Proton pump inhibitor or antacids—Last 3 months (n, %)	4 (12.9%)	0 (0.0%)	9 (25.7%)	0.058
Vegan or Vegetarian diet	1 (3.2%)	0 (0%)	0 (0%)	0.573

* n = 3 individuals (18.8%) developed active TB at follow-up. † n = 2 individuals (5.7%) developed active TB at follow-up. Statistical analysis were performed with analysis of variance (ANOVA) for continuous variables, and Fisher’s exact or Chi-squared test for categorical variables. Statistical significance was considered when p-values < 0.05 (two-tailed).

### Bacterial relative abundance in the nasopharynx of HHCs

**Figure 1:**
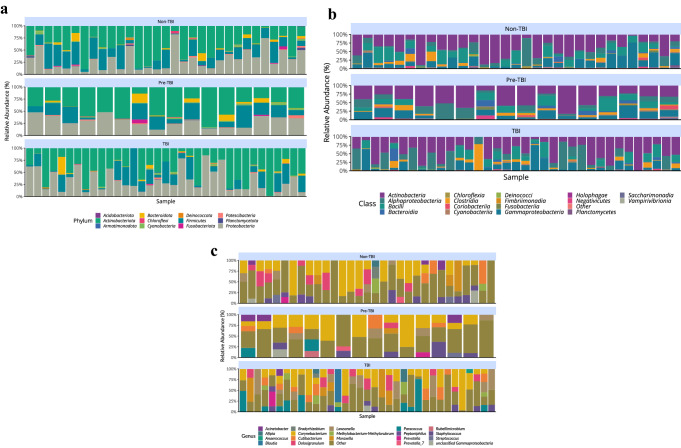
Taxonomic classification of the nasopharynx microbiota in TB exposed household contacts (n = 82). Descriptive individual variability of relative abundance according to LTBI uninfected (non-TBI), new LTBI on follow-up (pre-TBI) and LTBI from baseline evaluation (TBI) groups at (**a**) Phylum, (**b**) Class and (**c**) Genus level.

After dataset cleaning and filtering, 841 ASVs remained for evaluation. A descriptive analysis of relative abundance showed that for all HHCs, a total of 14 phyla were found, with the most predominant being: *Actinobacteriota* (43.8%), *Proteobacteria* (31.2%), *Firmicutes* (19.6%), and *Bacteroidota* (2.5%) (Table 2). There was variability in the predominance of less abundant phylum within each group (Supplementary Table S2) and a high degree of individual variability within groups (Fig. 1a). At the class level, for all HHCs, a total of 19 bacterial classes were found, dominating *Actinobacteria* (43.4%), *Alphaproteobacteria* (15.7%), *Gammaproteobacteria* (15.6%) and *Bacilli* (13.7%) (Table 3). Class domination varied for less abundant classes within each group (Supplementary Table S3) and a high individual variability within groups was also observed (Fig. 1b). At the genus level, the most frequently found were *Corynebacterium* (25%), *Cutibacterium* (7%), *Lawsonella* (6.9%), *Dolosigranulum* (6.1%), *Paracoccus* (5.7%) and *Staphylococcus* (5.5%). Groups exhibited a high degree of individual variability within each one (Fig. 1c). The *Mycobacterium* genus was not detected by 16S analysis in any of the analyzed participants, nor the bacteria itself by culture of the contralateral nasopharyngeal swabs.

**Table 2:** Percentage distribution at phylum level for all nasopharyngeal samples analyzed after amplicon sequence variant filtering (n = 82).

Phylum	Average (%)	Standard deviation (%)
Actinobacteriota	43.820	23.256
Proteobacteria	31.242	20.099
Firmicutes	19.557	17.273
Bacteroidota	2.530	5.351
Cyanobacteria	1.014	1.402
Patescibacteria	0.753	1.092
Fusobacteriota	0.348	1.173
Chloroflexi	0.257	0.686
Deinococcota	0.206	0.557
Planctomycetota	0.168	0.664
Acidobacteriota	0.082	0.198
Armatimonadota	0.025	0.140

**Table 3:** Percentage distribution at class level for all nasopharyngeal samples analyzed after amplicon sequence variant filtering (n = 82).

Class	Average (%)	Standard deviation (%)
Actinobacteria	43.402	23.343
Alphaproteobacteria	15.651	19.288
Gammaproteobacteria	15.590	15.856
Bacilli	13.701	13.554
Clostridia	5.253	9.608
Bacteroidia	2.530	5.351
Cyanobacteriia	0.969	1.363
Saccharimonadia	0.753	1.092
Negativicutes	0.603	1.573
Coriobacteriia	0.394	1.471
Fusobacteriia	0.348	1.173
Chloroflexia	0.257	0.686
Deinococci	0.206	0.557
Planctomycetes	0.168	0.664
Holophagae	0.082	0.198
Vampirivibrionia	0.044	0.196
Fimbriimonadia	0.025	0.140
Acidimicrobiia	0.023	0.097

In addition, we also compared the relative abundance of the ASVs classified at the species level. Although the length of the analyzed region is too short to distinguish among all present microbial species, some unique matches based on the region analyzed can be distinguished (Supplementary Fig. S2). Among the taxa present, there are some taxa prevalent in a large fraction of the samples in all groups, such as *Cutibacterium granulosum* (6.1%). Some species were more prevalent (present in at least 50% of the samples) in some of the groups. For example, *Dolosigranulum pigrum* (7.5%) was present in more than 50% of the samples of the non-TBI group. In contrast, *Kocuria rosea* (1.1%) was found in more than 50% of the samples of the TBI group (Supplementary Fig. S3).

### Bacterial diversity in the nasopharynx of HHCs

#### Alpha diversity

**Figure 2:**
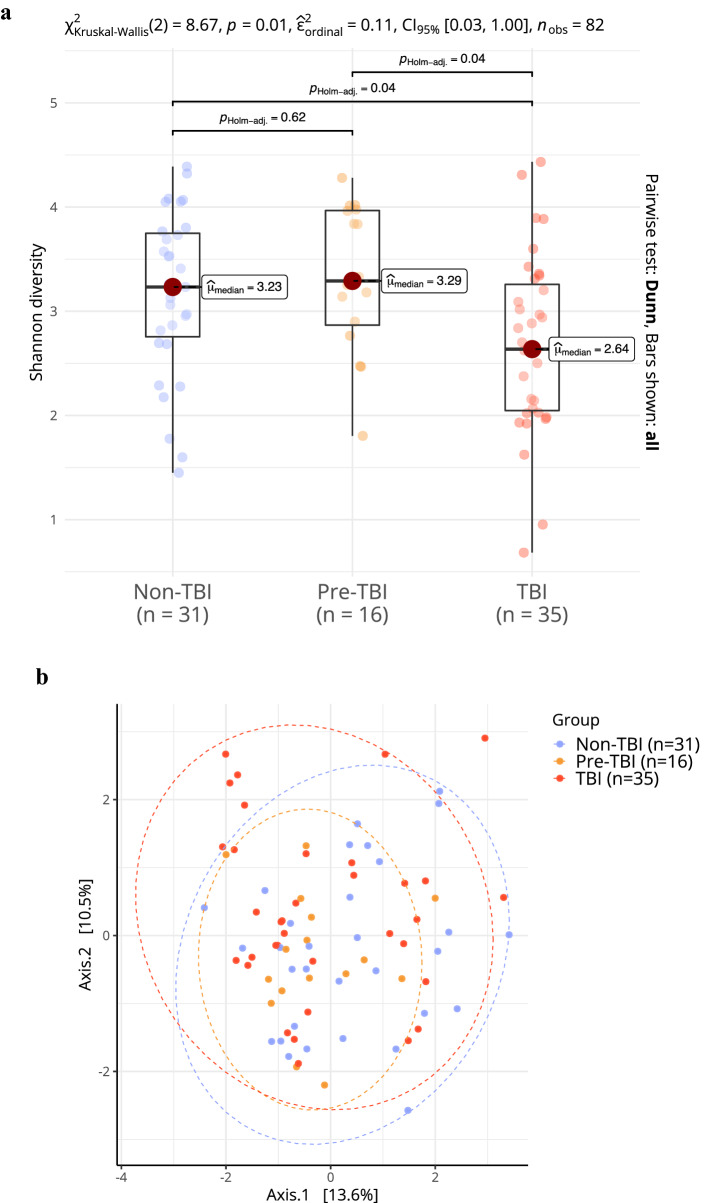
Changes in microbial diversity in the nasopharynx microbiota in TB exposed household contacts (n = 82). (**a**) Alpha diversity in HHCs samples using Shannon index. (**b**) Principal Coordinate Analysis plot showing the Euclidian distance of the PhilR-transformed data for each of the samples. Differences between group composition were significant (p = 0.036), only the comparison between non-TBI versus TBI (p = 0.035). Statistical analysis for alpha diversity was performed with Kruskal–Wallis test followed by Wilcoxon test with Holm adjustment for multiple testing. For beta diversity, a permutational analysis of variance based on the Euclidean distance matrix was performed.

Community diversity composition between HHCs groups was compared using the Shannon diversity index. The comparison showed significant differences between the group medians (non-TBI: 3.23 [2.76–3.75]; pre-TBI: 3.29 [2.87–3.97]; TBI: 2.64 [2.05–3.26], p = 0.01). Posthoc analysis using the Wilcoxon test showed statistical differences between the non-TBI vs. TBI group (p_adj_ = 0.04) and the pre-TBI vs. TBI group (p_adj_ = 0.04) (Fig. 2a). These results indicate that the microbiome of the participants with a latent TB infection had lower alpha diversity than the non-infected, including those non-infected but subsequently acquiring Mtb on follow-up, suggesting that structural differences may arise after acquiring Mtb.

#### Beta diversity

The microbial community composition comparison showed significant differences between groups (p = 0.036) (Fig. 2b) that the dispersion of the centroids could not explain within the dataset (p = 0.233). Pairwise comparison indicated only the communities between the non-TBI and the TBI group were different (p_adj_ = 0.035), while those of pre-TBI and TBI (p_adj_ = 0.095) and non-TBI and pre-TBI (p_adj_ = 0.189) were not. Thus, infected and uninfected individuals showed differences in the beta diversity of microbial communities, but the smaller group of those who later acquired the infection did not.

#### Core microbiome

**Figure 3:**
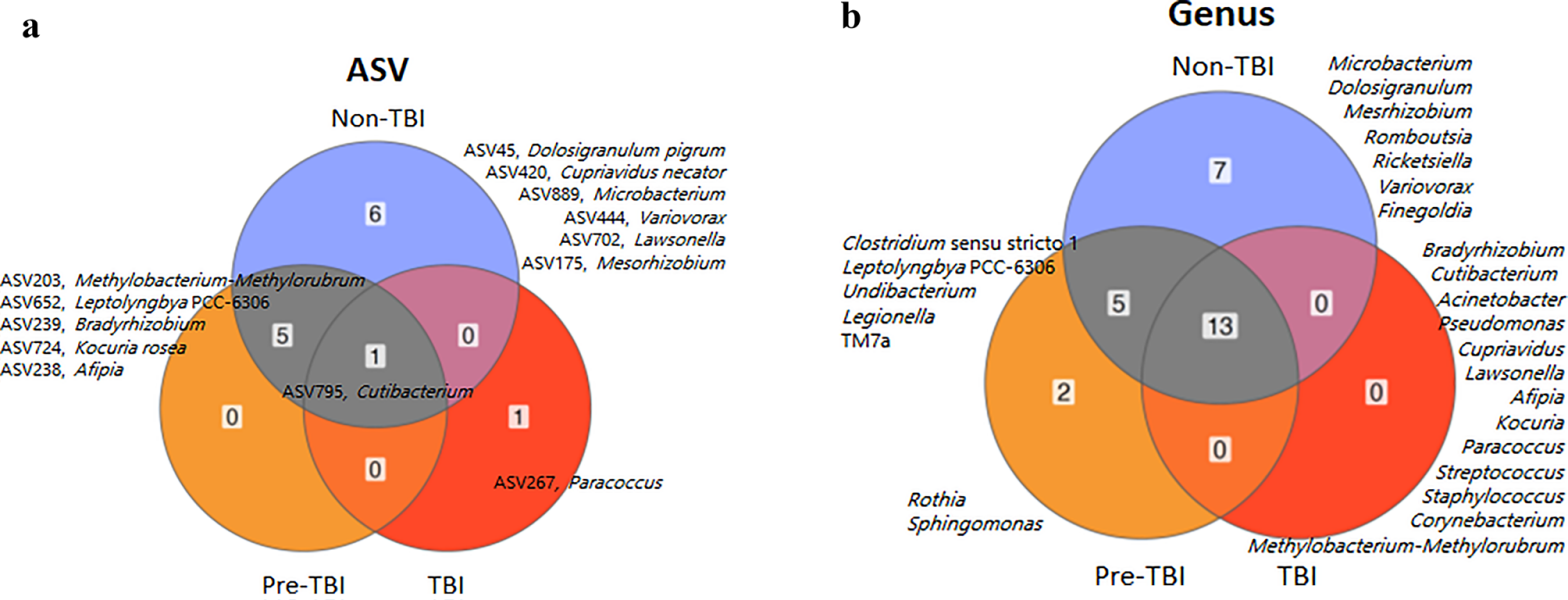
Core microbiome by TB infection status group, showing in each case ASVs or Genus that are present in at least 50% of the individuals within that group. (**a**) ASVs unique and shared among all groups. Names indicated the ASV number and its best taxonomic classification up to the species level. (**b**) Genus that are unique and shared between all groups. To identify taxonomic groups with statistically significant differences in abundance between groups was performed with the compositional approach implemented in ANCOMBC.

Evaluation of the core microbiome was performed for each HHCs group separately. At the ASV level, only a few ASVs were present in at least 50% of all samples from each group (non-TBI, 12 ASVs; pre-TBI, 6 ASVs; TBI, 2 ASVs). TBI and non-TBI, as well as TBI and pre-TBI, did not share any common ASVs. However, pre-TBI and non-TBI shared five ASVs (*Methylobacterium*-*Methylorubrum*, *Afipia*, *Bradyrhizobium*, *Leptolyngbya* PCC-6306, and *Kocuria rosea*), and TBI, pre-TBI and non-TBI shared only one (*Cutibacterium granulosum*) (Fig. 3a). Among unique ASVs in each group, six were associated with the non-TBI group (*Dolosigranulum pigrum*, *Mesorhizobium*, *Cupriavidus necator*, *Variovorax*, *Lawsonella* and *Microbacterium*), and 1 with the TBI group (*Paracoccus*).

At the genus level, we identified a core set of genera present in at least 50% of all samples within each group (non-TBI, 25; pre-TBI, 20; TBI, 13). There were 13 genera shared between the three of them (*Acinetobacter*, *Afipia*, *Bradyrhizobium*, *Corynebacterium*, *Cupriavidus*, *Cutibacterium*, *Kocuria*, *Lawsonella*, *Methylobacterium*-*Methylorubrum*, *Paracoccus*, *Pseudomonas*, *Staphylococcus* and *Streptococcus*), five between non-TBI and pre-TBI (*Clostridium *sensu stricto 1, *Legionella*, *Undibacterium*, *Leptolyngbya* PCC-6306, and TM7a), and none between pre-TBI and TBI, and non-TBI and TBI. Additionally, the non-TBI group had seven unique genera (*Finegoldia*, *Romboutsia*, *Dolosigranulum*, *Mesrhizobium*, *Ricketsiella*, *Variovorax* and *Microbacterium*). In contrast, the pre-TBI had only two (*Sphingomonas* and *Rothia*), and the TBI had none (Fig. 3b).

#### Differentially abundant genera between HHC groups

**Figure 4:**
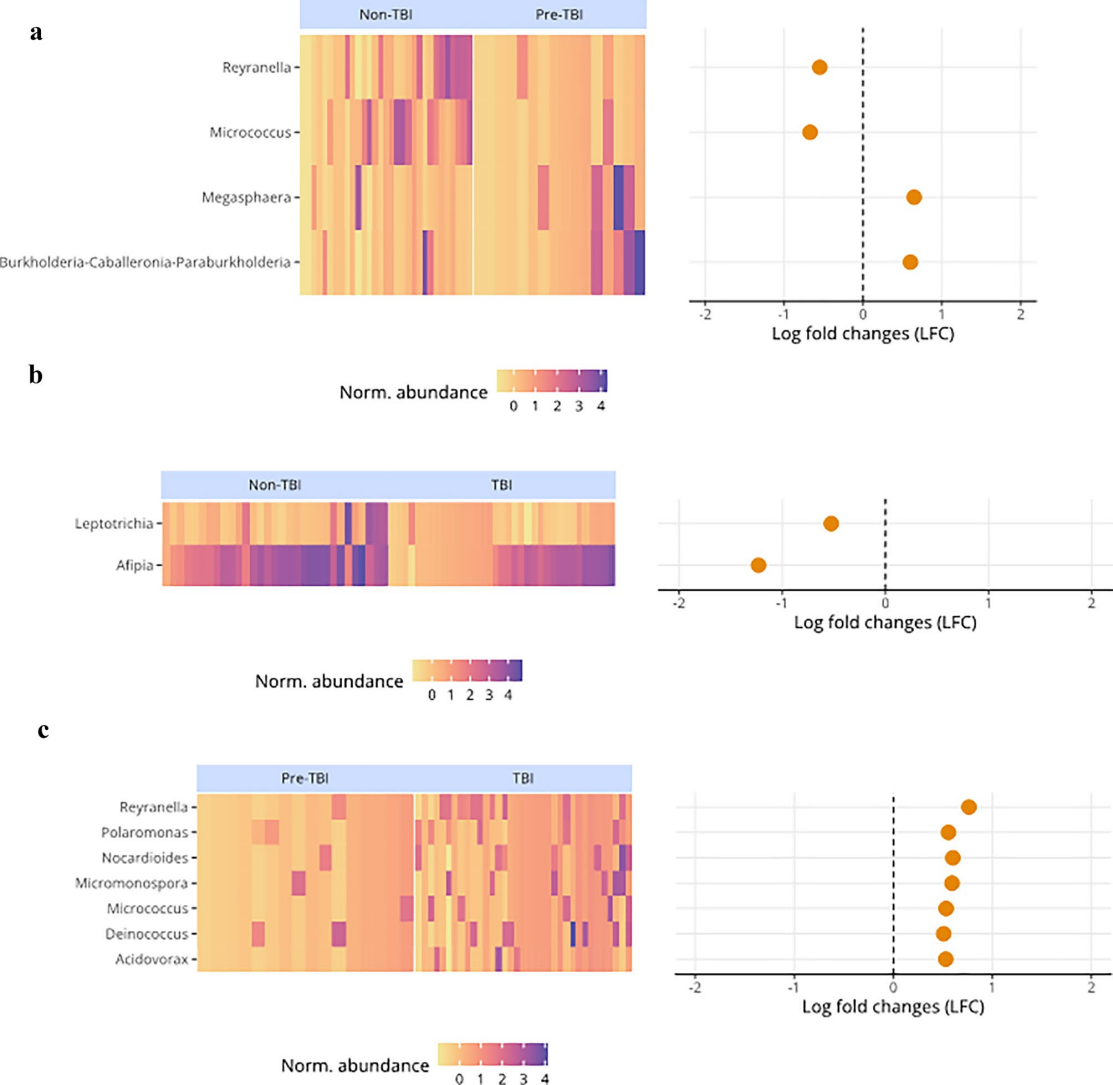
Genera with statistically significant differential abundance comparing household contacts (n = 82) TB infection status. (**a**) non-TBI vs. pre-TBI, (**b**) non-TBI vs. TBI, and (**c**) pre-TBI vs. TBI.

We used compositional-based analysis to evaluate differentially abundant genera between the three HHCs groups. Statistically significant differences (log fold changes) were found for non-TBI compared to pre-TBI, with *Reyranella* and *Micrococcus* being more abundant in the non-TBI group. At the same time, *Megasphaera* and *Burkholderia-Caballeronia-Paraburkholderia* were more abundant in the pre-TBI group (Fig. 4a). In addition, for non-TBI vs. TBI, *Leptotrichia* and *Afipia* were more abundant in the non-TBI group (Fig. 4b). For pre-TBI vs. TBI, seven genera, *Reyranella, Polaromonas, Nocardiodes, Micromonospora, Micrococcus, Deinococcus,* and *Acidivorax,* were more abundant in the TBI group (Fig. 4c). Finally, *Reyranella* was consistently lower in the pre-TBI dataset.

## Discussion

In the present study, we assessed the nasopharyngeal microbiome of a population of HHCs of patients with active TB. We found that individuals with an acquired latent TBI had a lower alpha diversity than uninfected individuals. Our results are consistent with reports of both nasal and oropharynx samples of patients with active PTB disease, who had a less diverse microbiota compared to healthy controls^17^, as well as the analysis of gut microbiota of children with PTB that reported a decreased microbial diversity^18^. Nonetheless, the findings observed in microbiota diversity in contacts who were about to acquire the infection remain to be confirmed in a larger cohort. Previous work by our group and others has reported that transient Mtb localization in the upper respiratory tract mucosa can rarely occur after exposure to a PTB case^19,20^. However, the persistence, replication and direct effect of Mtb in nasopharyngeal mucosa is unlikely, as we did not detect Mtb in the nasopharyngeal samples.

The interaction between intestinal microbial communities and the resident immune cells in the mucosa can lead to modulation of local and systemic immune response; an increased susceptibility to specific respiratory diseases (the “gut–lung axis”) has been proposed to develop when microbial dysbiosis occurs^21^. In murine models, broad-spectrum antibiotics-driven changes in the gut microbial composition affect the survival, burden, and dissemination of Mtb in the lungs, liver, and spleen compared to the mice with intact microbiota. Furthermore, fecal transplantation succeeds in restraining Mtb growth in the lungs preventing its dissemination^22^. Of interest, both macaques challenged with Mtb and household contacts having a latent TBI are less likely to progress to active TB when infected with *Helicobacter pylori*^23^. Although less explored, the upper and lower respiratory tract may also display immune system/microbiota interactions. In mice models, nasal instillation of *Staphylococcus aureus* attenuates influenza-mediated lethal inflammation in the lungs by inducing M2 alveolar macrophages^24^, and *Lactobacillus plantarum* administration protects against lethal pneumovirus infection in the lungs by TLR2 and NOD2 receptor signaling^25^.

The present study’s overall analysis of nasopharyngeal microbiota showed that for all HHCs, the predominant phyla were *Actinobacteriota*, *Proteobacteria*, *Firmicutes* and *Bacteroidota*. These results are consistent with other reports of the nasopharyngeal microbiota, with variations in their relative abundances^17,26–31^, and with microbiome studies of nasal and oropharynx samples from patients with active TB compared to uninfected individuals^17^. Interestingly, HHCs not acquiring latent TBI had a higher relative abundance mean (above 5%) of *Corynebacterium*, *Dolosigranulum*, *Cutibacterium* and *Staphylococcus*, similar to what is found in healthy non-exposed individuals^32^, while HHCs with an establish LTBI or those about to acquire a latent TBI had a higher relative abundance of *Corynebacterium*, *Lawsonella*, *Paracoccus*, *Cutibacterium*, *Dolosigranulum*, *Moraxella,* and *Corynebacterium*, *Staphylococcus*, *Cutibacterium*, *Lawsonella* and *Paracoccus*, respectively.

Our study is among the few that have analyzed the human nasopharyngeal microbiome in the latent TBI^33^ and, to our knowledge, the first to examine highly exposed HHCs from a prospective cohort acquiring or not Mtb infection at follow-up. However, there are several reports about gut^34,35^ and lungs’ microbiome characteristics of patients with established active PTB^17,36–39^, recurrent TB, and TB treatment failure^40^, with most studies including patients undergoing antimicrobial therapy. Regarding latent TBI, our findings differ from those of a study involving a comparatively small cohort size, in which the nasopharyngeal community diversity and microbial community composition of healthy controls (n = 18) were similar to that of latent TBI individuals (n = 19)^33^. These dissimilarities could lie in our control group that despite not having acquired the infection had being highly exposed to Mtb. Also, the differences could arise in the smaller number of patients included in that report and in the analytic approach, as the authors sequenced the V3-V4 region of the 16S rRNA gene and evaluated microbial diversity by looking at operational taxonomic units (OTUs) with a 97% or higher level of sequence similarity. In the current study, we sequenced the V1-V3 region which allows better resolution (even at the species level) for samples from human oral and respiratory tracts^41^, and also used ASVs, which allows for improved discrimination of ecological patterns^42^. Although not the scope of our study, the same authors found differences at the phylum and class levels between active TB and latent TBI, but not in alpha nor in beta diversity.

The lung microbiome resembles the healthy upper respiratory tract microbiome^10^, and whether a healthy microbiota in the nasopharynx can prevent Mtb establishment in the lower respiratory tract epithelium is unknown. Our study does not support this, as we did not find significant differences in microbiome composition (beta diversity) and relative abundance between HHCs acquiring a new Mtb infection at follow-up from those who remained uninfected. Notwithstanding, the core microbiome showed that the genus *Dolosigranulum* was one among which detection was only in the uninfected individuals. *Dolosigranulum* and its combination with *Corynebacterium* are a potential keystone species in the upper respiratory tract microbiota. Reports have shown an association with nasal health in chronic rhinosinusitis^43^, as well as a protective role in in vitro experiments against *Staphylococcus aureus*^43^ and *S. pneumoniae* colonization and otitis media in children^44,45^. This protective effect might be mediated by *Dolosigranulum’s* antimicrobial properties (anti-inflammatory and barrier-enhancing) by exploiting TLR2/6 interaction mechanisms within the host cells and L-lactic acid production^43^ or by its capacity to produce antibiotics or bioactive molecules that may play key roles in interspecies interactions with its microbial neighbors, e.g., for niche competition^46^. Both species are part of the healthy nasopharyngeal microbiota, even though *Dolosigranulum* has been rarely isolated from patients with bacteremia and queratitis^47^. Nonetheless, carriage surveys focused on detection of *S. pneumoniae* indicates the nasopharynx as the natural habitat of this bacterium^47^. Additionally, *Variovax*, also present only in non-infected HHCs, is a member of the human oral flora, has been previously identified as exclusive in nasal/oropharyngeal swabs of healthy participants compared to COVID-19 patients^48^, and it is also known for its strong biofilm formation^49^.

In our study, uninfected individuals who later acquired Mtb infection had a core microbiome that included the genus *Rothia*. Evidence shows that enrichment of lung microbiota with oral taxa such as *Rothia* induces host cellular mucosal immunity of the Th17/neutrophilic phenotype while suppressing innate immunity. Moreover, dysbiosis can predispose to or exacerbate conditions locally and at distal body sites^50^. We also found that individuals who later acquired Mtb showed an enrichment of the opportunistic pathogen *Megasphaera*, which has also been reported to be more abundant in the oropharyngeal microbiota of individuals with viral pulmonary infections as in COVID-19^51^. Taken together, we may speculate that the lack of *Dolosigranulum,* together with the presence *Rothia* and *Megasphaera* reflect a dysbiosis that might have rendered individuals more susceptible to Mtb infection.

The core’s microbiome present in at least 50% of Mtb infected individuals was represented only by *Paracoccus,* a gram-negative non-motile which genus, previously associated with infections, including leprosy and other skin infections where the skin microbiota had a relatively higher abundance of the bacteria compared to healthy controls^52,53^.

Among the present study limitations were the loss of samples due to low biomass recovery in the nasopharyngeal sampling process, leading to low DNA sequencing quality. Additionally, we found mild differences in participant's age that may have partially contributed to the observed differences between groups. Although, this does not seem to be clinically relevant as our participants were young (median age 34.5) and alterations in nasal microbiota communities start in middle-aged adults (40–65 years)^54^, similar to what has been found for gut microbiome that is relative stable in adults^55^. A further limitation of the study is that despite being embedded in a prospective cohort, the microbiome analysis was done only at the baseline visit (cross-sectional sampling), so spurious ecological associations cannot be ruled out. The study of samples at follow-up visits would have allowed assessment of changes in microbiome composition before and after Mtb acquisition as well as assessing whether it is the LTBI that induces changes in the microbiota composition or conversely, whether the upper respiratory tract microbiota structure confers susceptibility to acquiring this infection.

In conclusion, our study shows that HHCs with established latent TBI have differences in the nasopharyngeal microbiome with reduced alpha diversity and different bacterial communities compared to uninfected individuals. Whether an immune modulation of all respiratory microbiota ensuing Mtb infection occurs, as it has been described for active TB disease, remains to be established, as well as the protective role it might grant.

## Methods

### Patient selection

A prospective cohort study of household contacts (HHCs) of active pulmonary TB (PTB) cases (only acid-fast smear-positive index cases), aged 15 years or older (n = 231), was conducted between September 2017 and February 2020 in Santiago, Chile^56^. Contacts found to have active TB at baseline, reporting prior TB, autoimmune diseases, pregnancy, HIV infection, use of immunosuppressants, or inhalation drugs were not invited to participate. All HHCs were recruited within the first month after the TB diagnosis of the index case and screened for symptoms. Two nasopharyngeal swabs (one for 16S rRNA gene-targeted sequencing and another for Mtb culture) were taken at the baseline evaluation. Additionally, a chest X-ray to exclude secondary TB cases and an interferon-γ release assay (IGRA) test (QuantiFERON®-TB Gold Plus (QFT), QIAGEN, Hilden, Germany) were done at enrollment to evaluate TB infection (TBI) status. IGRA testing was repeated at a 12-weeks follow-up visit for all HHCs with negative IGRA results at the baseline evaluation, and participants were telephonically followed-up for 1 year to screen for symptoms of new active TB cases developing.

Participants were classified as: (a) “non-TBI” if the IGRA was negative both at baseline and at the 12-weeks evaluation, and they did not develop active TB on 1-year follow-up; (b) “pre-TBI” if the IGRA was negative at baseline evaluation but converted to positive IGRA at 12-weeks, or if the participant developed active TB on 1-year follow-up, and (c) “TBI” if the IGRA test was already positive at the baseline visit. Considering the known variability of the IGRA test around the positivity cut-off^57^, we included in the TBI category only HHCs with a positive IGRA result at baseline with CD4 or CD4-CD8 tubes IFN-γ values well above the positivity cut-off (≥ 1.0 UI/ml). Individuals who did not complete follow-up or had lower positive IGRA results (≤ 0.35 – 0.99 UI/ml) were excluded from the analysis (Supplementary Fig. S1).

### Ethical approval

The study has ethical approval from the Institutional Review Board of the Pontificia Universidad Católica de Chile. All eligible participants provided written informed consent. All methods were performed in accordance with the relevant guidelines and regulations.

### Nasopharyngeal sample collection

Nasopharyngeal samples were collected with Flocked swabs (Copan®, California, United States). For 16S sequencing, samples were stored in 2 ml of DNA preservative solution tubes (Norgen Biotek Corp., Ontario, Canada), kept on ice and then stored within 1 to 4 h of collection at − 80 °C until processing. Only one nasopharyngeal swab sample per participant—collected at the baseline visit—was analyzed. For Mtb culture, samples were stored in 2 ml PBS tubes and kept at room temperature until processing.

### 16S rRNA gene sequencing

Frozen swabs were thawed at room temperature and vortexed. Total DNA extraction of 500 μl was performed using the Saliva DNA Isolation Kit (Norgen Biotek Corp., Ontario, Canada) according to the manufacturer's supplementary protocol instructions at Instituto de Ciencias Biomédicas, Universidad de Chile. Frozen samples were shipped and sequenced at the Genomics Center of the University of Minnesota (Minneapolis, United States). Briefly, the genomic DNA (gDNA) content was quantified via qPCR of the 16S rRNA gene and used to normalize the gDNA concentration samples before library preparation. Libraries were prepared by amplifying the V1-V3 region of the 16S rRNA gene with 25 PCR cycles using the Nextera sequencing kit (Illumina, California, United States). Sequencing was performed with MiSeq 600 cycle v3 kit.

### Data processing and analysis

#### Amplicon sequencing variant generation

Sequencing data were processed with the DADA2 package^58^ in R version 4.2.0^59^ to generate amplicon sequencing variants (ASVs). Sequence quality was manually inspected to define trimming parameters. For sequence filtering and trimming, DADA2 function *filterAndTrim* was used with the following parameters: trimLeft = c(20,18), truncLen = c(290,275), maxEE = c(5,7), maxN = 0, truncq = 0. Error rates were estimated from the total dataset with the function *learnErrors* and the following parameters: max_consist = 20, nbases = 1e8. ASVs were generated with the *dada* function in pseudo-pooling mode. Read pairs were merged using the *mergePairs* function with minOverlap = 10 and maxMismatch = 1. Finally, the removal of chimeric sequences was done with the removeBimeraDenovo function using a consensus approach. The resulting ASVs were annotated using the *assignTaxonomy* function of DADA2 against the SILVA 138 database^60^. A phylogenetic tree was constructed for all ASVs by aligning them using MAAFT version 7.475^61^ and FastTree version 2.1.10^62^ using a General Time Reversible (GTR) model.

#### ASV analysis

##### ASV filtering

ASVs were removed if present in two different set control (preservation buffer and two water controls) used for library construction before sequencing. After ASVs generation, samples with less than 1,000 reads were also removed in a second filtering stage, as well as any ASV within the dataset classified as either contaminant (*Chloroplast, Mitochondria* or *Eukaryota*) or taxa with low prevalence (1 ASV in 1 sample at the Phylum level – *Abditibacteriota, Campylobacterota, Cloacimonadota*, *Entotheonellaeota, Elusimicrobiota*, *Halanaerobiaeota, Hydrogenedentes, Methylomirabilota, Nitrospinota, Nitrospirota,* SAR324 clade, *Sumerlaeota, Synergistota,* WOR-1, WS2, and WPS-2), keeping only ASVs present in at least four samples (5% of the dataset).

##### Microbiome analysis, alpha and beta diversity

Composition analysis of the cleaned dataset (841 ASVs) was done using *phyloseq*^63^ and its visualization with the *ggplot2* package^64^*.* Core microbiome analysis was performed using the *microbiome* package^65^. A core microbiome for each group (non-TBI, pre-TBI and TBI) was generated using a detection threshold of 0.001 and a prevalence of 50% of the samples within the group at the ASV and genus classification level.

For alpha diversity analysis, Shannon values were calculated using *phyloseq*^63^ for each sample. A Kruskal–Wallis test followed by a Wilcoxon test with Holm adjustment for multiple testing was applied to assess pairwise differences. Analysis and visualization were performed using the *ggstatsplot* package^66^.

Beta diversity was estimated using the compositional data (raw counts) and processed with the *PhILR* package^67^ to normalize counts with an isometric log-ratio transformation using the phylogenetic tree information (distance of the ASVs present in the samples). Sample distance was calculated on the transformed dataset with Euclidean distance using the *phyloseq* package^63^. To evaluate statistical differences between group clustering, a permutational analysis of variance was performed on the Euclidean distance matrix using the *adonis* function of the *vegan* package^68^. The variance homogeneity assumption was evaluated using the functions *betadisperser* and *permutest* of the *vegan* package.

##### Differentially abundant taxa

The compositional approach implemented in ANCOMBC^69^ that allows for compositional analysis of the microbiome data with bias correction was used to identify taxonomic groups with statistically significant differences in abundance between HHCs groups. This method acknowledges sources of variation in microbiome datasets, including unequal sampling fractions and differences in sequencing efficiency, to attempt to limit the effects of group unevenness due to the microbiome variability among individuals in our study. The ASVs were summarized at the genus level, and all three pairwise comparisons of the groups (non-TBI vs. TBI, non-TBI vs. pre-TBI, and pre-TBI vs. TBI) were performed using default parameters. Multiple hypothesis testing was corrected with the Benjamini–Hochberg method with a false discovery rate of 0.05; the resulting differentially abundant ASVs were filtered, keeping only those with a log fold change of at least 0.5.

### Statistical analysis

Clinical and demographic data analyses were performed in RStudio^59,70^. Comparisons between groups were performed with analysis of variance (ANOVA) for continuous variables, and Fisher’s exact or Chi-squared test for categorical variables. Statistical significance was considered when p-values < 0.05 (two-tailed).

## Supplementary Information

Supplementary Information 1.

Supplementary Information 2.

Supplementary Information 3.

## Data Availability

The datasets generated and/or analyzed during the current study are available in the European Nucleotide Archive (ENA), Project Accession number PRJEB61078.
