# Supplementary material for: Reduced microbial diversity of the nasopharyngeal microbiome in household contacts with latent tuberculosis infection
Source: Sci Rep. 2023 May 5;13:7301. doi: 10.1038/s41598-023-34052-8 (PMC10160714; doi:10.1038/s41598-023-34052-8)
Supplement: Supplementary file 1 — Supplementary Information 1. [file 41598_2023_34052_MOESM1_ESM.docx]

Title: Reduced microbial diversity of the nasopharyngeal microbiome in household contacts with latent tuberculosis infection.

Authors:

Cinthya Ruiz-Tagle^1^, Juan A. Ugalde^2^, Rodrigo Naves^3^, Rafael Araos^4,5^, Patricia García^6^, and María Elvira Balcells^1*^.


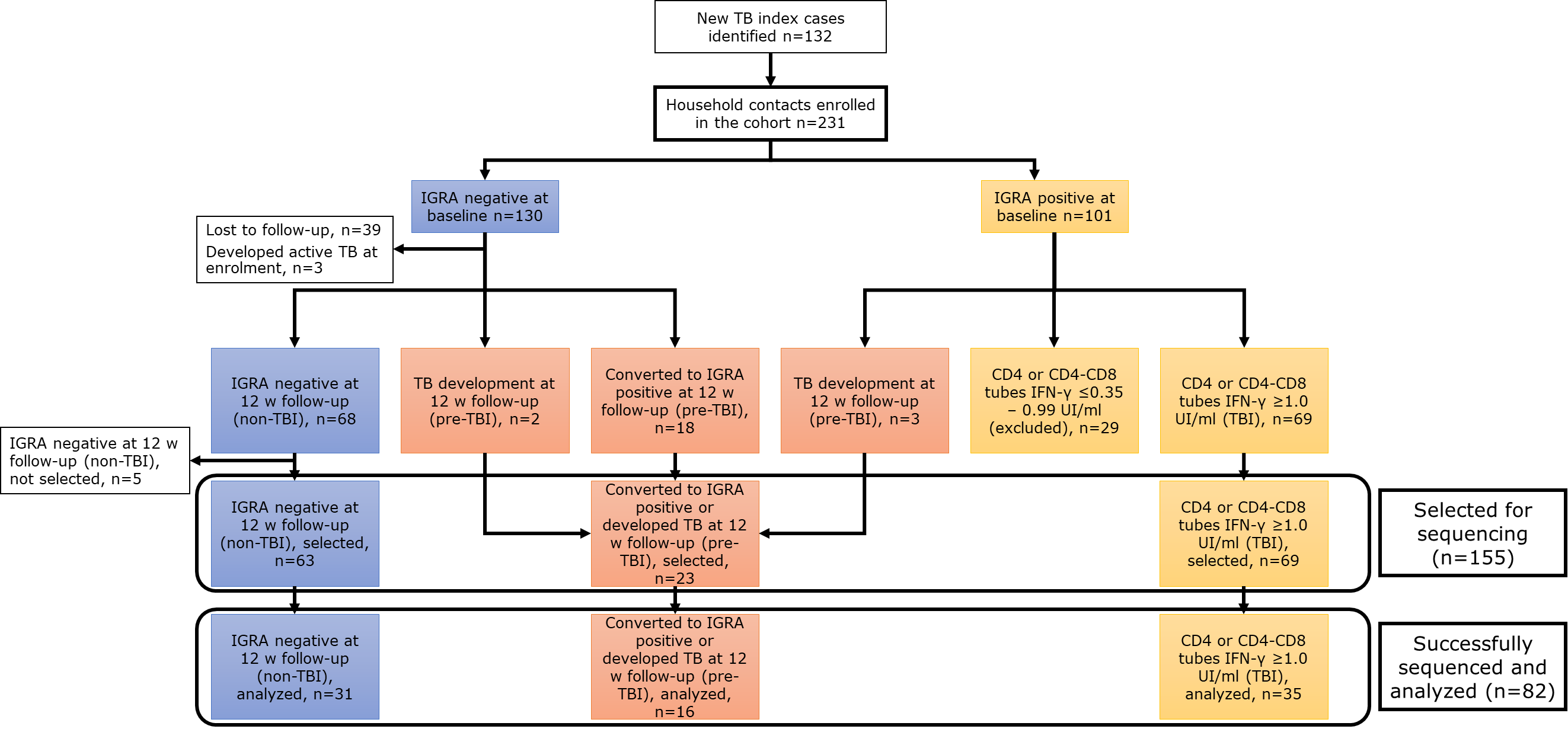


Supplementary Figure S1. Study flowchart.

Supplementary Figure S2. Relative abundance of species identified in TB exposed household contacts. The heatmap illustrates the relative abundance of species identified in the different groups (non-TBI, pre-TBI, and TBI). Only species with an abundance higher than 1% were included.

Supplementary Figure S3. Shared species in over 50% of samples in TB exposed household contacts. The heatmap shows species shared in over 50% of samples within each group (non-TBI, pre-TBI, and TBI) as average relative abundance with the associated standard deviation for each species within the groups.

Supplementary Table S1. Clinical and epidemiological characterization of TB contacts from the entire cohort study (n=231).

|  | **Excluded***  **(N=76)** | **Unsuccessfully sequenced (N=73)†** | **Successfully sequenced and analyzed (N=82)‡** | **p-value** |
| --- | --- | --- | --- | --- |
| Female sex (n, %) | 35 (46.1%) | 40 (54.8%) | 48 (58.5%) | 0.276 |
| Mean age, years (SD) | 38.4 (15.7) | 33.8 (13.1) | 34.5 (13.7) | 0.107 |
| Countries, total (n, %) |  |  |  |  |
| Chile | 29 (38.2%) | 23 (31.5%) | 18 (22.0%) | 0.083 |
| Other Latin American countries | 47 (61.8%) | 50 (68.5%) | 64 (78.0%) |  |
| Tobacco smoker (n, %) | 22 (29.3%) | 9 (12.3%) | 21 (25.6%) | 0.088 |
| Self-reported viral respiratory infections in last month (n, %) | 26 (34.2%) | 28 (38.4%) | 28 (34.1%) | 0.827 |
| Any antibiotic use - Last 6 months (n, %) | 9 (11.8%) | 12 (16.4%) | 13 (15.9%) | 0.685 |
| Probiotic or vitamin use - Last 3 months (n, %) | 14 (18.4%) | 14 (19.2%) | 12 (14.6%) | 0.721 |
| Proton pump inhibitor or antacids - Last 3 months (n, %) | 14 (18.4%) | 9 (12.3%) | 13 (15.9%) | 0.589 |
| Vegan or Vegetarian diet | 1 (1.3%) | 0 (0%) | 1 (1.22%) | 1 |

* 3 individuals (3.9%) developed active TB at baseline. † 1 individual (1.4%) developed active TB at baseline and 3 (4.1%) at follow-up. ‡ n=5 individuals (6.1%) developed active TB at follow-up. In the entire cohort: n=4 individuals (1.7%) developed active TB at baseline and 8 (3.5%) at follow-up. Statistical analysis were performed with analysis of variance (ANOVA) for continuous variables, and Fisher’s exact or Chi-squared test for categorical variables. Statistical significance was considered when p-values <0.05 (two-tailed).

Supplementary Table S2. Phylum ranking of abundance and percentage distribution (%) with standard deviation (SD) for all household contacts groups analyzed after amplicon sequence variant filtering (n=82).

|  | **Non-TBI**  **(n=31)** | | **Pre-TBI**  **(n=16)** | | **TBI**  **(n=35)** | |
| --- | --- | --- | --- | --- | --- | --- |
|  | **Ranking** | **% (SD)** | **Ranking** | **% (SD)** | **Ranking** | **% (SD)** |
| **Acidobacteriota** | 11 | 0.120 (0.251) | 9 | 0.119 (0.248) | 12 | 0.031 (0.078) |
| **Actinobacteriota** | 1 | 42.174 (23.506) | 1 | 44.425 (18.904) | 1 | 45.001 (25.283) |
| **Armatimonadota** | 12 | 0.016 (0.055) | 12 | 0.013 (0.050) | 11 | 0.038 (0.207) |
| **Bacteroidota** | 4 | 2.316 (4.124) | 4 | 3.737 (5.510) | 4 | 2.168 (6.240) |
| **Chloroflexi** | 9 | 0.356 (1.010) | 8 | 0.233 (0.404) | 8 | 0.180 (0.366) |
| **Cyanobacteria** | 5 | 1.015 (1.474) | 5 | 1.616 (1.438) | 5 | 0.737 (1.267) |
| **Deinococcota** | 10 | 0.253 (0.688) | 10 | 0.061 (0.147) | 7 | 0.230 (0.546) |
| **Firmicutes** | 3 | 22.396 (16.501) | 3 | 18.503 (14.295) | 3 | 17.523 (19.190) |
| **Fusobacteriota** | 7 | 0.419 (1.165) | 7 | 0.715 (2.054) | 9 | 0.118 (0.314) |
| **Patescibacteria** | 6 | 0.874 (0.978) | 6 | 1.159 (1.654) | 6 | 0.460 (0.781) |
| **Planctomycetota** | 8 | 0.369 (1.039) | 11 | 0.037 (0.146) | 10 | 0.050 (0.169) |
| **Proteobacteria** | 2 | 29.692 (20.385) | 2 | 29.385 (11.663) | 2 | 33.463 (22.953) |

Supplementary Table S3. Class ranking of abundance and percentage of distribution (%) with standard deviation (SD) for all household contacts groups analyzed after amplicon sequence variant filtering (n=82).

|  | **Non-TBI**  **(n=31)** | | **Pre-TBI**  **(n=16)** | | **TBI**  **(n=35)** | |
| --- | --- | --- | --- | --- | --- | --- |
|  | **Ranking** | **% (SD)** | **Ranking** | **% (SD)** | **Ranking** | **% (SD)** |
| **Acidimicrobiia** | 17 | 0.026 (0.064) | 15 | 0.048 (0.193) | 18 | 0.010 (0.042) |
| **Actinobacteria** | 1 | 41.854 (23.665) | 1 | 43.254 (19.120) | 1 | 44.842 (25.266) |
| **Alphaproteobacteria** | 4 | 11.555 (15.380) | 3 | 14.392 (12.598) | 2 | 19.855 (23.944) |
| **Bacilli** | 3 | 17.029 (13.952) | 4 | 12.650 (11.471) | 4 | 11.233 (13.818) |
| **Bacteroidia** | 6 | 2.316 (4.124) | 6 | 3.737 (5.510) | 6 | 2.168 (6.240) |
| **Chloroflexia** | 12 | 0.356 (1.010) | 12 | 0.233 (0.404) | 11 | 0.180 (0.366) |
| **Clostridia** | 5 | 4.795 (6.166) | 5 | 4.367 (3.846) | 5 | 6.064 (13.365) |
| **Coriobacteriia** | 13 | 0.295 (1.322) | 10 | 1.122 (2.629) | 12 | 0.148 (0.495) |
| **Cyanobacteriia** | 7 | 0.952 (1.426) | 7 | 1.588 (1.401) | 7 | 0.703 (1.230) |
| **Deinococci** | 14 | 0.253 (0.688) | 14 | 0.061 (0.147) | 9 | 0.230 (0.546) |
| **Fimbriimonadia** | 18 | 0.016 (0.055) | 18 | 0.013 (0.050) | 15 | 0.038 (0.207) |
| **Fusobacteriia** | 10 | 0.419 (1.165) | 11 | 0.715 (2.054) | 13 | 0.118 (0.314) |
| **Gammaproteobacteria** | 2 | 18.137 (17.003) | 2 | 14.993 (10.515) | 3 | 13.608 (16.880) |
| **Holophagae** | 15 | 0.120 (0.251) | 13 | 0.119 (0.248) | 17 | 0.031 (0.078) |
| **Negativicutes** | 9 | 0.572 (1.251) | 8 | 1.486 (2.860) | 10 | 0.227 (0.624) |
| **Planctomycetes** | 11 | 0.369 (1.039) | 16 | 0.037 (0.146) | 14 | 0.050 (0.169) |
| **Saccharimonadia** | 8 | 0.874 (0.978) | 9 | 1.159 (1.654) | 8 | 0.460 (0.781) |
| **Vampirivibrionia** | 16 | 0.063 (0.243) | 17 | 0.028 (0.076) | 16 | 0.034 (0.191) |
