# Supplementary figures and images for: Reduced microbial diversity of the nasopharyngeal microbiome in household contacts with latent tuberculosis infection
Source: Sci Rep. 2023 May 5;13:7301. doi: 10.1038/s41598-023-34052-8 (PMC10160714; doi:10.1038/s41598-023-34052-8)

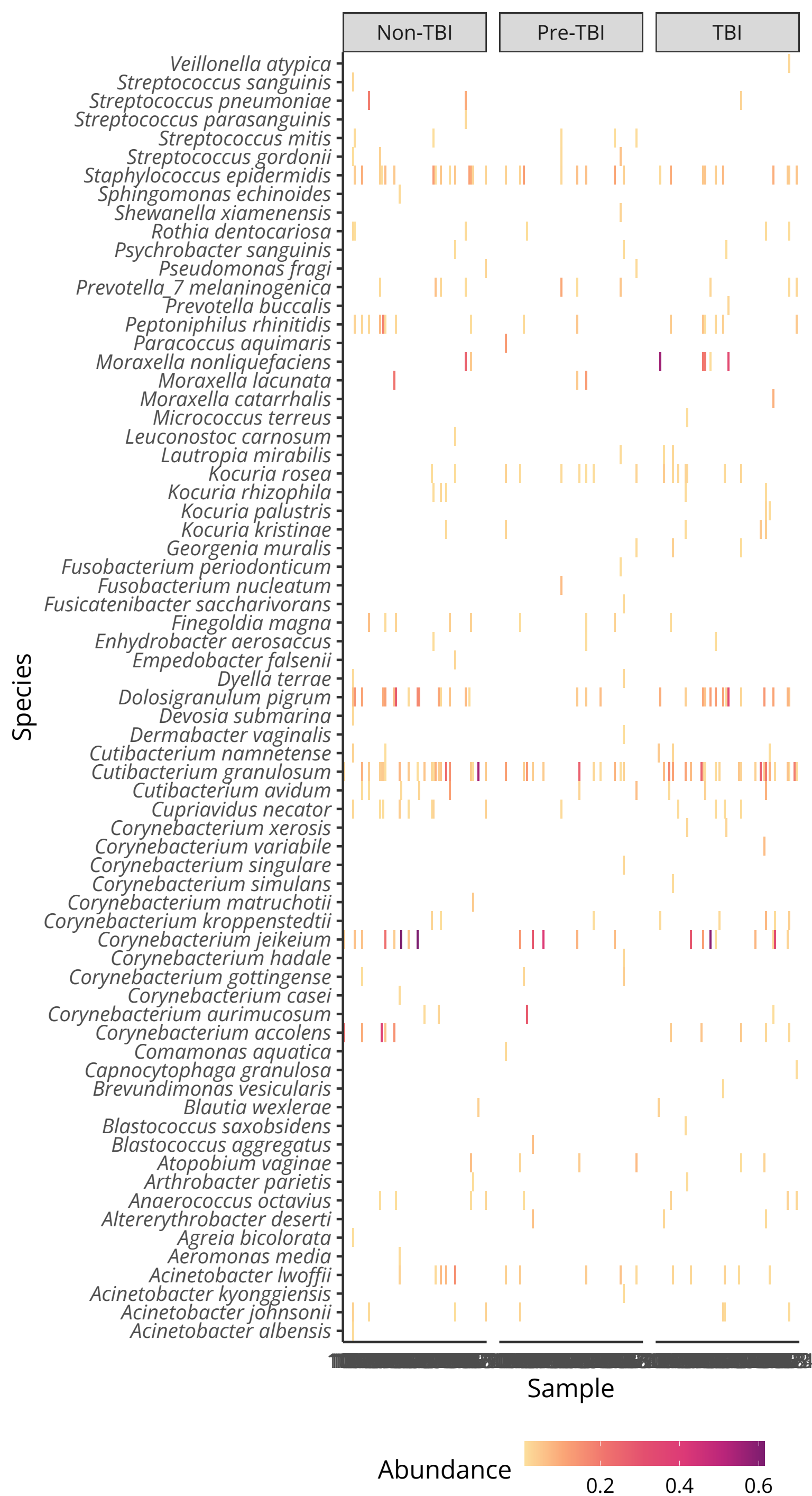

Supplement: Supplementary file 2 — Supplementary Information 2. [file 41598_2023_34052_MOESM2_ESM.pdf]

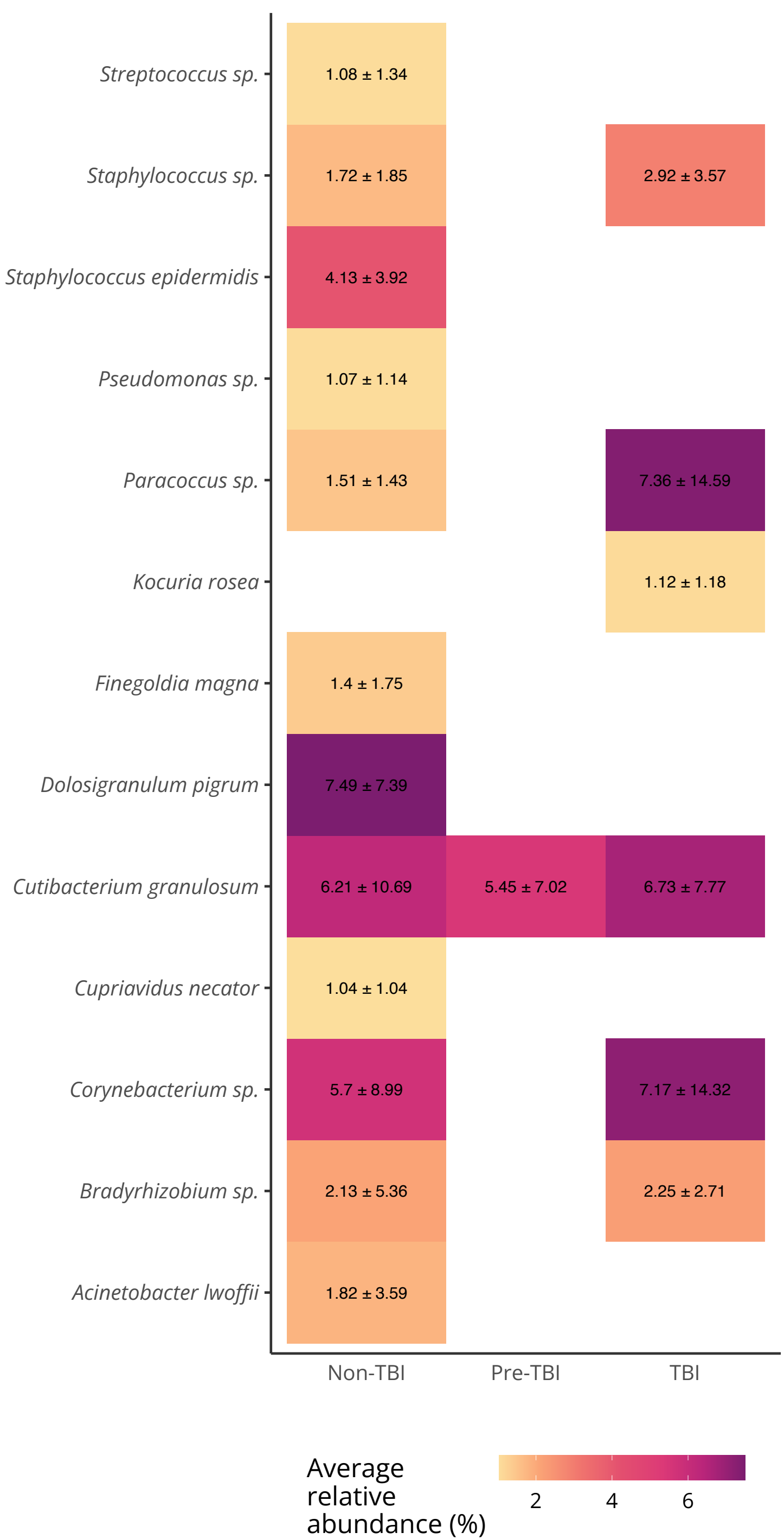

Supplement: Supplementary file 3 — Supplementary Information 3. [file 41598_2023_34052_MOESM3_ESM.pdf]
